# Pharmacological Evaluation of the Anticancer Activity of Extracts and Fractions of *Lannea barteri* Oliv. (Anacardiaceae) on Adherent Human Cancer Cell Lines

**DOI:** 10.3390/molecules25040849

**Published:** 2020-02-14

**Authors:** Florence N. Mbaoji, Steven Behnisch-Cornwell, Adaobi C. Ezike, Chukwuemeka S. Nworu, Patrick J. Bednarski

**Affiliations:** 1 Department of Pharmacology and Toxicology, Faculty of Pharmaceutical Sciences, University of Nigeria, Nsukka, PMB 410001, Enugu State, Nigeria; adaobi.ezike@unn.edu.ng (A.C.E.); chukwuemeka.nworu@unn.edu.ng (C.S.N.); 2 Institute of Pharmacy, Pharmaceutical and Medicinal Chemistry, University of Greifswald, 17487 Greifswald, Germany; steven.behnisch@uni-greifswald.de

**Keywords:** *Lannea barteri*, leaf, stem bark, adherent human carcinoma cell lines, 5637, KYSE 70, SiSo, HepG2

## Abstract

In western Africa ethnomedicine, *Lannea barteri* Oliv. (Anacardiaceae) is believed to have activity against gastrointestinal, neurological and endocrine diseases. Previous studies on this plant have revealed antimicrobial, anticholinestrase, anticonvulsant, antioxidant and anti-inflammatory activities. However, the anticancer potential of *L. barteri* has not been studied to date. The aim of this study was to evaluate the anticancer potential of hot and cold extracts and silica gel column chromatographic fractions of *L. barteri* leaf and stem bark. The extracts and fractions were tested for anticancer activity by using the crystal violet cell proliferation assay on four adherent human carcinoma cell lines—5637 (bladder), KYSE 70 (oesophagus), SiSo (cervical) and HepG2 (hepatic). The inhibitory concentration (IC_50_) of fractions IH, 1I, 2E and 2F were: 3.75 ± 1.33, 3.88 ± 2.15, 0.53 ± 0.41, and 0.42 ± 0.45 µg/mL against KYSE 70 and 1.04 ± 0.94, 2.69 ± 1.17, 2.38 ± 3.64, 2.17 ± 1.92 µg/mL against SiSo cell lines respectively. Fraction 2E showed weak apoptotic activity at double the IC_50_ and some sign of cell cycle arrest in the G2/M phase. Thus, phytoconstituents of *L. barteri* leaf and stem bark can inhibit the proliferation of cancer cell lines indicating the presence of possible anticancer agents in this plant.

## 1. Introduction

Medicinal plants used in folk medicine are a rich source of compounds with anticancer activities. Studies of traditional medicine have resulted in pharmacological entities that have good efficacy and different mechanisms of action from the existing orthodox medicines. It is believed that phytoconstituents are good alternatives to conventional drugs used in the treatment of diseases because they are inexpensive and may have less adverse effects [1]. Despite the growing concern about these plant products due to their lack of standardization, they are in high demand by traditional care givers for the treatment of various diseases including cancer. Thus, there are growing research interests geared towards natural products for finding new drug entities from among the myriad of plant species in African forests that are largely untapped. These efforts have often been successful since many plant materials have been found to have numerous and diverse pharmacological activities. *Lannea barteri* is one of such plant that may be a source of anticancer agents.

**Figure 1:**
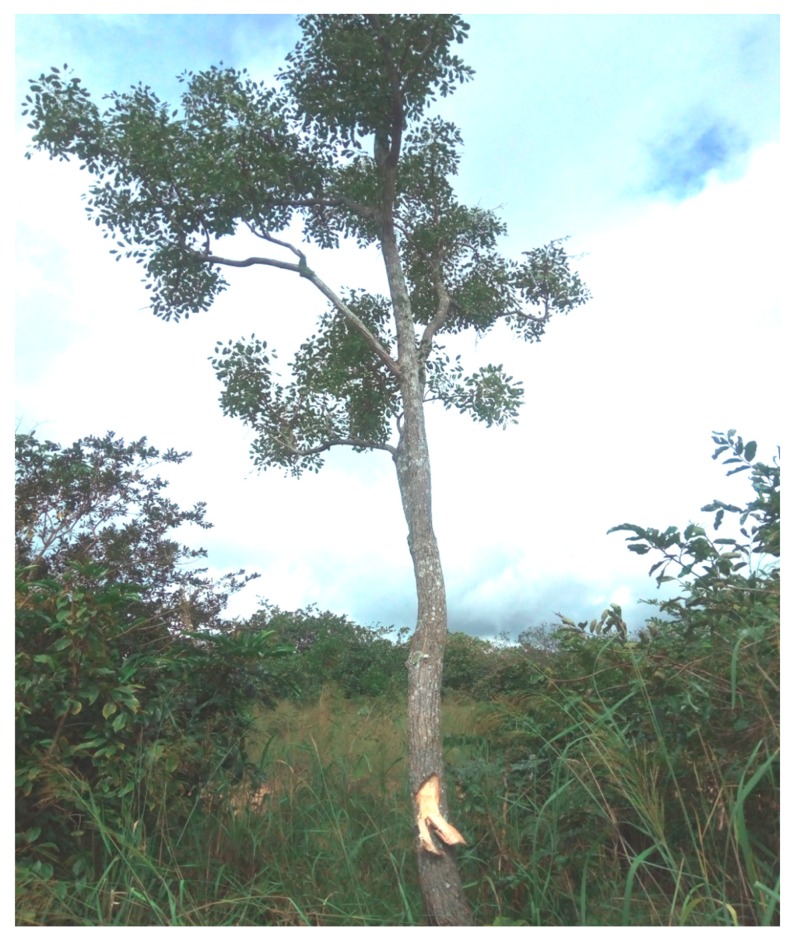
*Lannea barteri* tree growing in Nigeria used in sample collection.

*L. barteri* is a dioecious tree with a spreading crown; it can grow from 5–18 meters tall (Figure 1). The bole is usually straight and clear of branches for several meters. It can be up to 40 cm in diameter with a thick bark. Leaves are alternate, pinnately compound with 2–6 pairs of opposite leaflets and a terminal cone, rachis 10–25 cm long, leaflets shortly stalked. Flowers are unisexual, regular, pedicel up to 3 mm long, calyx-cupped shaped with lopes 1 mm long, ciliate, petals oblong yellowish with darker veins. Fruits are compressed cylindrical drupe 10–13 mm × 7–8 mm glabrous, purplish red [2].

Indeed *L. barteri* is widely used in western Africa in folk medicine. It is called sinsabega in Bimoba, mon in Dahomey Bassari, kuntunkuni in Ghana Akan-Asante, kuntunkurfi in Brong and in Ivory Coast Baule it is called kondro. It is known as faruhi in the northern part of Nigeria, and tiuko in Guinea [3]. *L. barteri* originates from East Guinea and is distributed in Ethiopia, Uganda, South of DR Congo, Nigeria, Mali, Burkina Faso, Ghana, Cote d’ Ivoire and Burundi [4,5]. The bark is frequently used as a stomachic. A decoction of the root is drunk as a treatment for gastric pains, diarrhoea, oedema, paralysis, epilepsy and madness [4]. When combined with the bark of *Lannea kerstingii*, it is used as a vermifuge [4]. The bark is used externally to treat ulcers, sores and leprosy. The root decoction is taken to cure hernias. The root is ground, wrapped in the leaves of an unknown species, and applied as poultice on wounds. A leaf decoction is taken to cure haemorrhoids [6]. Phytochemical investigations on the stem bark and root of the plant have revealed the presence of saponins, coumarins, polyphenols, alkaloids, tannins, quinones, steroids, terpenoids, and flavonoids [7,8]. Previous works on the stem bark and root bark of *L. barteri* have revealed antimicrobial [7,8], anticholinesterase, DPPH radical scavenging [7], anticonvulsant [9] activities, and on leaf extract antioxidant, hepatoprotective [10], wound healing and anti-inflammatory activities [11]. Oxidative stress and chronic inflammatory response have deleterious effect on macromolecules; thus, they can trigger different types of cancers. Since the antioxidant and anti-inflammatory activities of this plant extract are good properties for cancer prevention, and considering the link between oxidation and chronic inflammation to the development of cancer, the need to explore the anticancer activity of this plant extract arose. The aim of this study was to evaluate the anticancer potential of hot and cold extracts and column chromatography fractions of leaf and stem bark of *L. barteri* on four adherent human carcinoma cell lines—5637 (bladder), KYSE 70 (oesophagus), SiSo (cervical) and HepG2 (hepatic) as well as the toxic effect of the extracts on *Artemia salina* larva.

## 2. Results

### 2.1. Yield of Extracts and Fractions 

The extraction processes yielded hot (h) and cold (c) dichloromethane (DCM) (DLEh, DLEc, DSbEh, DSbEc), methanol (MLEh, MLEc, MSbEh, MSbEc) and water (WLEh, WLEc, WSbEh, WSbEc) extracts. The yields of the various extracts are shown in Table 1. The thin layer chromatography (TLC) of DLEc and DSbEc gave different spots with varying retention factors (Table 2). Similarly, fractionation of the DLEc and DSbEc by silica gel column chromatography yielded 11 fractions (1A–1K) from DLEc and 10 fractions (2A–2J) from DSbEc (Table 3).

### 2.2. Phytochemical Constituents of Extracts and Most Active Chromatography Fractions

Phytochemical analysis on the DLEc and DSbEc gave positive reactions for terpenoids and steroids, respectively.

### 2.3. The RP-HPLC Chromatograms of the Active Fractions from the Cold DCM Extracts of Leaf (1E–1J) and Stem Bark (2D–2I) 

The chromatograms of the active fractions from the cold DCM extracts of leaf (1E–1J) and stem bark (2D–2I) revealed absorption peaks (Au) of different heights at varying wavelengths between (210-600 nm) and retention times. The chromatogram of fraction 1E revealed five prominent peaks between retention times 24 and 32 min (Figure S1). Fraction 1F gave eight peaks with two prominent peaks occurring at 29.20 and 31.01 min (Figure S2). Fraction 1G gave a total of six peaks with three major sharp peaks at 28.72, 29.19 and 30.96 min retention times (Figure S3). Fraction 1H gave nine major peaks with the first three peaks well separated from one another at retention times of 13.25, 14.83 and 16.61 min; also, the eighth peak was very sharp at retention time of 39.37 min (Figure S4). Fraction 1I had two major peaks occurring at retention times of 28.72 and 30.64 and clusters of other peaks (Figure S5). Fractions 1J and 1K had similar peaks with two major peaks occurring at 20.73/20.64 min and 30.73/30.56 min respectively and with clusters of other minor peaks (Figures S6 and S7).

Fraction 2D gave three peaks with one sharp peak which eluted at 24.75 min (Figure S8). Fraction 2E gave five peaks, two sharp peaks and other broad peaks (Figure S9). Fraction 2F gave three prominent and well–separated sharp peaks at 18.96, 23.73 and 31.87 min with two additional minor peaks (Figure S10). Fraction 2G gave seven peaks some of which were well separated (Figure S11) while fraction H gave two broad peaks and one sharp peak which occurred at 27.41 min (Figure S12). Fraction 2I gave only one broad peak that eluted at 14.67 min (Figure S13).

### 2.4. Effect of Extracts of on 5637, KYSE 70 and SiSo Cancer Cell Lines Proliferation in Primary Screening

DLEh and DSbEh inhibited the proliferation of all the cancer cell lines while the MLEh, MSbEh, WLEh and WSbEh showed no growth inhibition. The (T/C) _corr_. (%) values of DLEh, MLEh, MSbEh, WLEh and WSbEh for the various cancer cell lines are shown in Table 4.

DLEc and DSbEc extracts inhibited the proliferation of all the cancer cell lines tested while MLEc and MSbEc inhibited 5637 and SiSo cell lines only. The WLEc and WSbEc did not inhibit the proliferation of any of the cancer cell lines. The T/C _corr_. (%) values for the extracts that had activity are shown in Table 4.

The order of antiproliferation activity of DLEc and DSbEc on the cell lines was SiSo> KYSE 70 > 5637.

The extracts with T/C _corr_. values less than 50% were thus selected for secondary screening.

### 2.5. Effects of Extracts on KYSE 70 and SiSo Cell Lines in Secondary Screening

The IC_50_ values of the hot extracts (DLEh, DSbEh. MLEh and MSbEh) against the KYSE 70 and SiSo cell lines are shown in Table 5. The DSbEh inhibited growth of both cell lines more than the DLEh. The MLEh inhibited only the SiSo cell line while the MSbEh inhibited the growth of none of the cancer cell lines (Table 5).

The growth inhibitory effect of the DSbEc on the KYSE 70 and SiSo were significantly (*p* < 0.05) higher than that of DLEc. The MLEc significantly (*p* < 0.05) inhibited the SiSo cell line more than the MSbEc (Table 5).

**Figure 2:**
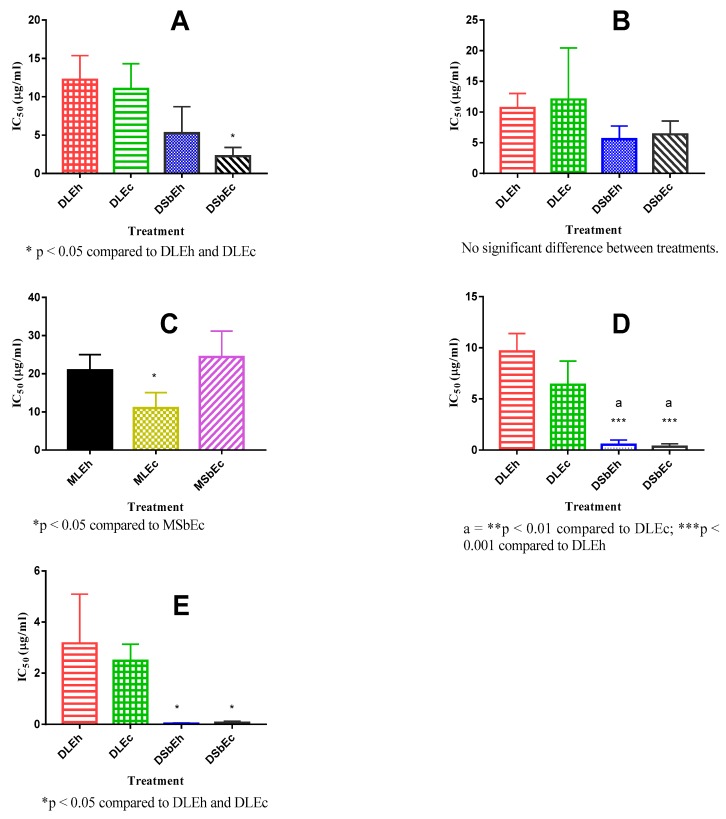
Comparison of IC_50_ of hot and cold DCM and methanol extracts of leaf and stem bark against KYSE 70, SiSo, HepG2 and 5637 cell lines. (**A**) = KYSE 70 cell line, (**B**,**C**) = SiSo cell line, (**D**) = HepG2 cell line, (**E**) = 5637 cell line. Values are mean ± SEM (*n* = 3) one-way ANOVA. (Tukey’s multiple comparisons test). DLEc—cold DCM leaf extract, DSbEh—hot DCM stem bark extract, MLEc—cold methanol leaf extract, MSbEc—cold methanol stem bark extract, MLEh—hot methanol leaf extract.

The comparison of the cell proliferative inhibitory activity of the hot and cold DCM extracts on the KYSE 70 cell line showed no significant difference between DLEh and DLEc, and between DLEh, DLEc and DSbEh; but there was significant (*p* < 0.05) difference between DLEh, DLEc and DSbEc with DSbEc showing the highest inhibitory activity (Figure 2A). There was no significant difference in the growth inhibitory activities of SiSo cancer cell line by the leaf and stem bark extracted with hot and cold DCM (Figure 2B). 

There was no significant difference in the growth inhibitory activity of MLEh, MLEc and MSbEc on SiSo cell line. MLEc produced significantly (*p* < 0.05) higher inhibitory activity on SiSo cancer cell line when compared to MSbEc (Figure 2C). 

On the HepG2 cancer cell line, both the hot and cold DCM extracts of the leaf and stem bark inhibited the proliferation of this cell line (Figure 2D). DSbEh and DSbEc significantly (*p <* 0.001) inhibited the proliferation of HepG2 cancer cell line. When compared, DLEh and DLEc did not produce as much inhibition of HepG2 cancer cell line proliferation as DSbEh and DSbEc. Their IC_50_ values of the inhibition of the various cancer cells proliferations are shown in shown in Figure 2D.

The cold and hot DCM leaf and stem bark extracts inhibited the proliferation of 5637 with lower IC_50_ values (Figure 2E). The inhibitory activities against 5637 were significantly (*p* < 0.05) higher with DSbEh and DSbEc compared to DLEh and DLEc (Figure 2E).

### 2.6. Effect of Column Chromatography Fractions of DLEc (fractions 1A–K) and DSbEc (fractions 2A–I) on 5637, KYSE 70, SiSo and HepG2 Cell Lines Proliferation in Primary Screening

After screening the various extracts for anticancer activity, those extracts with biological activity (e.g., DLEc, DSbEc) were subject to silica gel column chromatography and the collected fractions again screened for anticancer activity. The results showed that fractions 1E and 2F produced a higher inhibitory effect on the proliferation of 5637 of cell lines than the other fractions tested (Table 6). Fractions 1H and 2E produced remarkable proliferation inhibition against KYSE 70 cell line (Table 6); fractions 1D and 2E inhibited the proliferation of SiSo cell line to a greater extent compared to the inhibition produced by other fractions (Table 6) while fractions 1H, 2D and 2H inhibited the proliferation of HepG2 cell line largely compared to other fractions (Table 6). 

### 2.7. Effect of Column Chromatography Fractions of DLEc (Fractions 1E–1K) and DSbEc (Fractions 2D–2I) on KYSE 70 and SiSo and HepG2 Cell Lines Proliferation in Secondary Screening 

**Figure 3:**
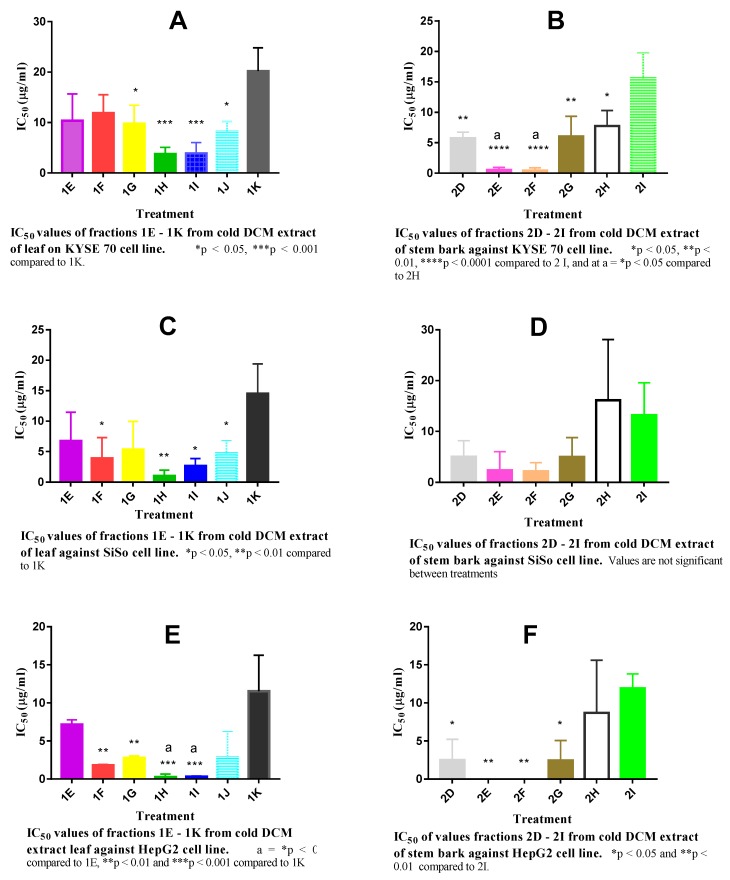
IC_50_ values of fractions 1E-1K and 2D–2I from DLEc and DSbEc respectively against the cell lines. (**A**,**B**) = KYSE 70 cell line, (**C**,**D**) = SiSo cell line and (**E**,**F**) = HepG2 cell line. Values are mean ± SEM (*n* = 3) one-way ANOVA (Tukey’s multiple comparisons test).

Secondary screening was done on the various active fractions to estimate IC_50_ values. The IC_50_ value of fractions 1H (3.75 µg/mL) and 1I (3.88 µg/mL) were significantly (*p* < 0.001) lower than that of fraction 1K (20.21 µg/mL) when inhibition of cell proliferation produced by all the fractions against KYSE 70 cell line were compared (Figure 3A). Similarly, the IC_50_ values of fractions 1G (9.77 µg/mL) and 1J (8.22 µg/mL) were significantly lower than that of fraction 1K while the IC_50_ values of fractions 1E (10.37 µg/mL) and IF (11.89 µg/mL) were lower than that of fraction K but not significantly (Figure 3A). 

The IC_50_ values in µg/mL for these fractions were 5.77 (2D), 0.53 (2E), 0.42 (2F), 6.05 (2G), 7.76 (2H) and 15.58 (2I). There was significant (*p* < 0.0001) difference between the IC_50_ of fractions 2E and 2F compared to fraction 2I on their inhibition against KYSE 70 (Figure 3B). There was significant (*p* < 0.01) difference in the IC_50_ values of fractions 2D and 2G in comparison to that of fraction 2I while the IC_50_ of fraction 2F significantly (*p* < 0.05) differed from that of fraction 2H just as the IC_50_ of fraction 2H significantly (*p <* 0.05) differed from that of fraction 2I (Figure 3B). 

The leaf fractions inhibited the proliferation of SiSo cell line to varying extent. Significant (*p* < 0.05) inhibition of SiSo cell line proliferation were recorded with fractions 1F, 1H, 1I, 1J and (*p <* 0.01) with fraction 1H compared to fraction 1K (Figure 3C). 

There was no significant difference in the IC_50_ values of the stem bark fractions that inhibited the proliferation of SiSo cell line when compared with the various treatments (Figure 3D).

The proliferative inhibitory activity of fractions 1H and 1I against HepG2 cell line were significant (*p* < 0.05 and *p <* 0.001) when compared to fractions 1E and IK respectively; they had the lowest IC_50_ values of 0.25 and 0.30 µg/mL respectively when all the treatment groups were compared (Figure 3E). 

Fractions 2E and 2F had the lowest IC_50_ values (8.8 × 10^−4^ and 2.06 × 10^−4^µg/mL, respectively) when compared with other fractions (2D, 2G, 2H, and 2I) against HepG2. The inhibitory effect of the 2E and 2F fractions against the proliferation of HepG2 cell line were significant (*p* < 0.01) compared to fraction 2I (Figure 3F).

### 2.8. Effect of Column Chromatography Fractions 1H, 1I 2E, 2F on Apoptosis Induction in SiSo Cancer Cell Line

**Figure 4:**
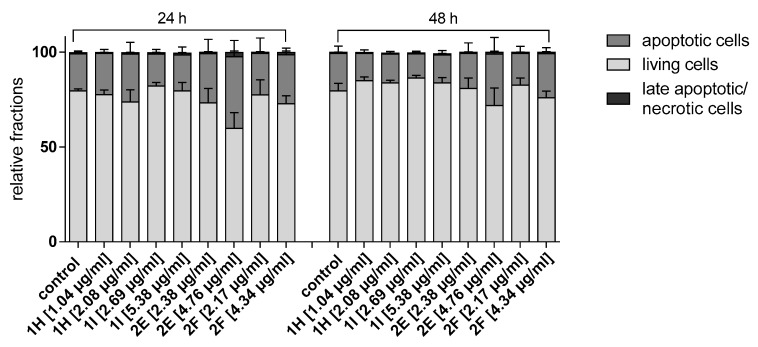
Effect of column chromatography fractions 1H, 1I 2E, 2F (at IC_50_ concentration and doubled the IC_50_ concentration) on induction of apoptosis in SiSo cancer cell line after 24 and 48 h incubation periods.

The percentage of apoptotic cells induced by the fractions and DMSO at IC_50_ value after 24 h and 48 h incubation periods respectively were 22.34%, 15.51% (DMSO), 19.9%, 16.38% (1H), 18.52%, 11.58% (1I), 31.09%, 11.74% (2E), 30.87% and 15.30% (2F) (Figure 4). At concentrations doubled the IC_50_ values, the percentage of apoptotic cells induced by the fractions and DMSO at 24 h and 48 h incubation periods were 20.25%, 16.79% (DMSO), 31.16%, 14.19% (1H), 19.95%, and 17.25% (1I), 47.07%, 34.00% (2E), 27.85% and 25.83% (2F) respectively. From this result, fraction 2E gave the highest fractions (47.07% and 34.00%) of apoptotic cells at doubled the IC_50_ concentration after 24 h and 48 h incubation periods, respectively, compared with other fractions and control. Meanwhile, the leaf fractions especially fraction 1I had the highest number (0.68 and 1.22%) of necrotic cells than the stem bark fractions at doubled the IC_50_ concentration after those incubation periods.

### 2.9. Effect of Fractions 1H, 1I 2E, 2F on Cell Cycle Progression in SiSo Cancer Cell Line

**Figure 5:**
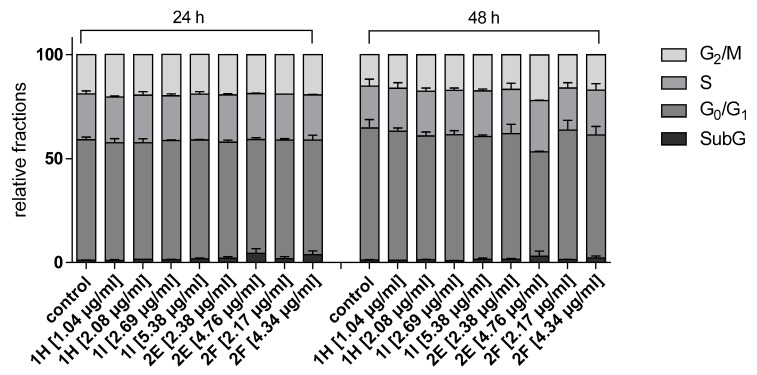
Effect of column chromatography fractions 1H, 1I 2E, 2F on cell cycle in SiSo cancer cell line.

**Figure 6:**
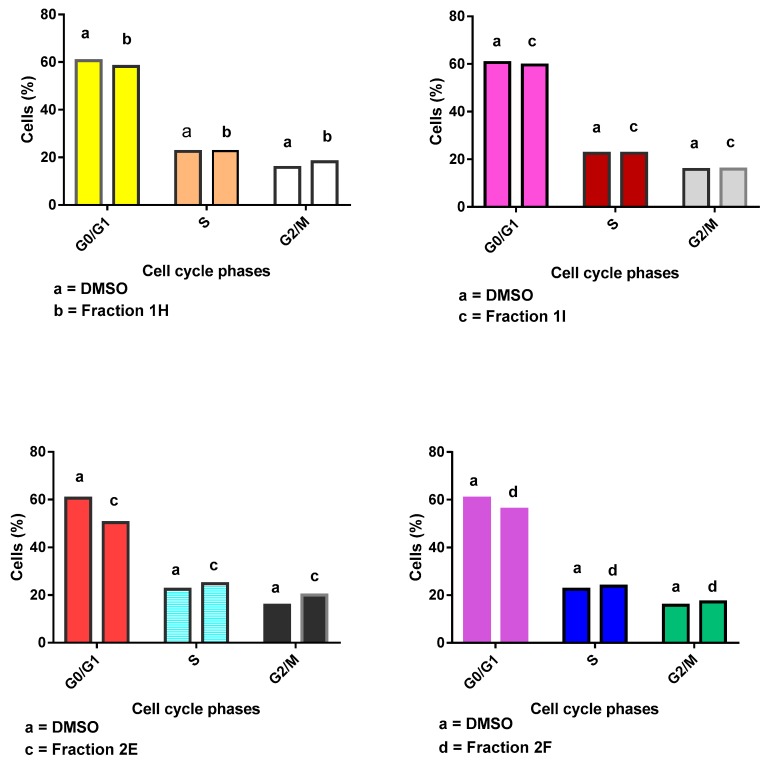
Effect of fractions column chromatography 1H, 1I, 2E, and 2F at doubled the IC_50_ concentration on cell cycle progression in SiSo cancer cell line after 48 h incubation.

In the cell cycle measurement, there was a non-significant reduction in the population of cells (50.39%) in the G1/G0 phase with concomitant increase in the population of cells accumulated in S (24.78%) and G2/M (20.04%) phases especially at doubled the IC_50_ concentration of fraction 2E at 48 h incubation period compared to control (Figure 5 and Figure 6). Fraction 1H accumulated the highest percentage (21.28%) of cell population at G2/M phase followed by fraction 1I (20.47%) at doubled the IC_50_ value after 24 h incubation period eventhough these changes were not significant (Figure 5 and Figure 6).

### 2.10. Toxic effect of Extracts on Brine Shrimp 

To test for general toxicity of the extracts *in vivo*, the brine shrimp assay was used. In this assay, the lethal concentration at 50% of the population (LC_50_) of the DLEc was found to be 5649.20 µg/mL while the extrapolated effective concentration was 564.92 µg/mL. However, the LC_50_ of DSbEc was 124.78 µg/mL while the EC_50_ was 12.48 µg/mL. At 1000 µg/mL, the percentage death was high (66.7%) with DSbEc and low (13.3%) with DLEc. The calculated therapeutic index (TI) for both extracts was 9.99 (Table 7).

## 3. Discussion 

Cancer is a life-threatening and difficult-to-manage disease that ultimately overwhelms the entire bodily functions of the patient. This disease requires a multi-disciplinary approach to identify new pharmacological entities that will result in success and give strong and positive hope to the patient and the care giver. Some cancers can be cured completely when detected early either with chemotherapeutic agents, surgery, ionization therapy or a combination of two or more of these treatment options. Over the years, researchers have searched for new lead pharmacological compounds either from plants or other biological products that can be better than the existing drugs in terms of efficacy and safety. Certain plants have been investigated for their anticancer potentials and some of the plant materials have been found effective as anticancer agents. One such plant that interested us is *Lannea barteri,* a plant native to some African countries. This study investigated the anticancer potential of *L. barteri* leaf and stem bark extracts and fractions on four adherent human cancer cell lines: KYSE 70, SiSo, 5637 and HepG2 by using the crystal violet cell proliferation assay as a bioactivity guide [12]. Furthermore, the cytotoxic effect of DLEc and DSbEc on brine shrimp and the mechanism of anticancer activity of the fractions were studied. The phytoconstituents - terpenoids and steroids were present in DLEc and DSbEc, respectively.

Proliferation in cells is favored when there is phosphorylation of specific regulatory proteins by cyclin–dependent kinases, the engine that makes the cell cycle run [13]. Adherent cells detach from cell culture plate during cell death and this characteristic could be used for the indirect quantification of cell death and to find differences in proliferation upon stimulation with death-inducing agents [14]. Simple screening methods to detect maintained adherence of cells are the staining of the attached cells with either crystal violet, neutral red or sulforhodamine B dyes. Crystal violet binds to proteins and DNA. When cells undergo cell death they detached from cell culture plate and the amount of crystal violet staining is reduced [15]. Indeed, some of the extracts and fractions with cytotoxic activity were able to reduce the color intensity of crystal violet, showing inhibition of cell proliferation and marked cell death at low concentrations (< 30 µg/mL).

Primary screening of plant materials for anticancer activity with the crystal violet assay is a standard method for selecting choice pharmacological products for secondary screening to enable testing at range of doses [12]. The primary screening was designed to assess the threshold for cell growth inhibition produced at one concentration of extract (e.g., 50 µg/mL), enabling only extracts which satisfy the threshold inhibition criteria to progress to the 5-dose screening designed to capture efficiently the compounds with antiproliferative activity in the form of IC_50_ values [16].

The results of primary screening revealed that the DCM and methanol extracts were cytotoxic to the cell lines at concentrations below 50 µg/mL and thus were subjected to secondary screening. 

*L. barteri* hot and cold extracts and silica gel chromatography fractions showed strongest antiproliferative activity against KYSE 70, SiSo, 5673 and HepG2 cancer cell lines at very low IC_50_ values. The DSbEh and DSbEc gave more inhibition against all the cell lines than DLEc while the MLEc inhibited 5637 and SiSo cell lines more than the MSbEc. This activity increased with column fractionation of the extracts. There was a selective activity of extract and fractions from the stem bark for the KYSE 70 cell line more than those from the leaf, suggesting its efficacy in oesophageal squamous cell carcinoma [17]. 

SiSo is a human cancer cell line which was established from a patient afflicted with uterine cervical adenocarcinoma. The growth of SiSo cell line was markedly inhibited by the cold dichloromethane extracts which increased with fractionation. The DLEc and its fractions had more inhibitory effect on SiSo and high selectivity for this cell line than other extracts and fractions. 

Brine shrimp lethality assay is an essential tool used for preliminary assessment of toxicity of medicinal plant products by measuring their potentials to kill larvae of *Artemia salina* leach [18]. It is an all-or-none method of assay. The lethal concentration (LC_50_) of the DLEc that killed the 50% of the population of the larvae was higher than the concentration of DSbEc that killed the same percentage of the population. The concentration of the DLEc effective at 50% of the population (EC_50_) was higher than that of DSbEc even though they had the same therapeutic index. The EC_50_ of a quantal dose response curve represents the concentration of a compound where 50% of the population exhibits a response after a specific exposure time. It also measures the potency of substances [19]. Therefore, the stem bark extract with lower EC_50_ and LC_50_ values was more potent as toxic agent than the leaf extract.

Assessment of the mechanism of cytotoxic activity of the most active fractions from the leaf and stem bark extracts of *L. barteri* revealed some level of apoptotic activity and cell cycle arrest. Apoptosis is a form of programmed cell death in multicellular organisms that involves biochemical cascade of events leading to morphological changes such as (blebbing, cell plasmolysis, nuclear fragmentation, chromatin condensation and chromosomal DNA fragmentation) and eventual death without affecting the normal cell [20]. Apoptosis can be initiated by anticancer drugs, gamma and ultraviolet irradiation, deprivation of survival factors such as interleukin-1, and several other cytokines that activate “death receptors” such as Fas and tumour necrosis factor receptors [20]. Fraction 2E had higher apoptosis induction potential than other fractions. This was evident with the greater percentage of fractions of annexin V positive cells than those that are positive to both annexin V and propidium iodide (PI). Annexin V binds to phosphatidyl serine on the extracellular surface of the cell membrane while propidium iodide binds DNA of only cells that have lost their membrane integrity. However, living cells with intact cell membrane do not stain positive to PI, and cells that are positive to Annexin V and PI are considered dead while those only positive to Annexin V are considered to be undergoing apoptosis [21,22].

The cell cycle is a physiological process that occur in distinct stages to assure controlled cell growth, development and division. Cell proliferation and differentiation are under tight genetic control in normal cells. For cancer to arise there is altered control in the genetic machinery that controls cell proliferation and eventual progression of stages of cell cycle which is the hallmark of cancer growth and sustenance [23]. Cell cycle analysis by DNA content measurement is a method that most often employs flowcytometry to distinguish cells in different phases of the cell cycle and quantification of intracellular components especially DNA, as well as cell cycle-related metabolic molecules such as cyclin-dependent kinases and cyclins [23]. The fractions showed non-specific cell cycle arrest with fraction 2E showing the greatest accumulation of DNA at G2/M phase and corresponding decrease in G1/G0 phase in SiSo cancer cell line. This suggests that fraction 2E may be arresting the cell cycle progression at this stage implying that DNA damage may be partly involved in the cell death.

In conclusion, our findings show that the cold DCM extracts and fractions from the *Lannea barteri* leaf and stem bark possess potent anticancer activity against 5637 (urinary bladder), KYSE 70 (oesophageal squamous), SiSo (cervix adeno-) and HepG2 (hepatocellular) human carcinoma cell lines. The stem bark extract showed greater activity than the leaf extract. It would appear that apoptosis and cell cycle arrest at G2/M phase are likely anticancer mechanisms of action. The anticancer activity may be attributed to unknown phytoconstituents inherent in the plant. Work is ongoing to purify the most active fraction, isolate and characterize the compound(s) that have the anticancer activity, to test them on other cancer cell lines and to explore other possible mechanism(s) of cytotoxic activity.

## 4. Materials

### 4.1. Equipment and Apparatuses

Digital and electronic weighing balance (Satorius^®^ Entris, BP 21005, Goettigen, Germany), ultrasonicator (Bandelin Sonopuls UW 2070^®^, Berlin, Germany), magnetic stirrer (Heidolph models MR^®^ 3000 and MR 3001, Schwabach, Germany), rotary evaporator (Buchi Rotavapor^®^, R-200 and R-210, Duisburg, Germany) with heating bath (Buchi heating bath^®^, B-490 and B-491, ), folded qualitative filter paper 301 size 185 mm with particle retention size of 12–15 µm, flasks (conical and round bottom), measuring cylinders, mechanical grinding machine, refrigerator of−20°C, lyophilizer, analytical TLC plates (silica gel 60 F_254_, 20 × 20) and silica gel (Kieselgel 60 H, 70–230 mesh, Merck, Darmstadt, Germany), VLM EVA 2^®^ Evaporator (VLM GmbH, Bielefeld, Germany), column fractions retriever, 96–well microtiter plates and25 mL and 75 mL flasks (Sarstedt, Nümbrecht, Germany), 8- and 12- channel–pipette (Labopette 50–250 µL, Hirschmann^®^, Eberstadt, Germany), centrifuge (Heraeus Biofuge primo R) and incubator (Heracell, ThermoFisher Scientific, Waltham, M.A, USA), particle counter and size analyzer (Coulter Z2, Beckman Coulter, Fullerton, CA, USA), sterile filters (PAN^®^ Biotech GmbH, Aidenbach, Germany), universal shaker, (Lab Tec Edmund BühlerGmbH TiMix, Hechingen, Germany), HPLC system (Merck-Hitachi LaChrom, Fukuoka, Japan), microtiter plate reader (SPECTRAmax Plus, Molecular Devices, San Jose, CA, USA) and flow cytometer (MACS Quant Analyzer, Miltenyi Biotec, Teterow, Germany).

### 4.2. Chemicals and Solvents

All solvents were analytical grade and water was purified either by distillation or with an Elga PureLab Flex4 (Veolia Water Tech, Celle, Germany), penicillin/streptomycin solution (10,000U penicillin/mL, 10 mg streptomycin/mL), RPMI-1640 culture medium^®^ and L-glutamine (PAN Biotech, Aidenbach, Germany), ribonuclease-A (Rnase) and NaHCO_3_ (Carl Roth GmbH, Karlsruhe, Germany), 50% glutaraldehyde solution, trypsin—EDTA solution and fetal calf serum (FCS) (Sigma-Aldrich, Munich, Germany), HPLC gradient acetonitrile (ACN) (VWR International, Darmstadt, Germany), Dulbecco’s buffer (pH 7.4): KCl—0.2 g/L, NaCl—8 g/L, MgSO_4_.7H_2_O—0.1 g/L, KH_2_PO_4_—0.2 g/mL, Na_2_HPO_4_.7H_2_O—1.55 g/mL, AnnexinV apoptosis kit (Miltenyi Biotec, Teterow, Germany), propidium iodide (Applichem, Darmstadt, Germany), phosphate buffered saline (PBS), Triton X-100 (Merck, Darmstadt, Germany).

### 4.3. Cancer Cell Lines 

KYSE 70, SiSo, 5673 and HepG2 human cancer cell lines were obtained from the German Collection of Microorganisms and Cell Culture (DMSZ Braunschweig, Germany).

### 4.4. Brine Shrimp Larvae

*Artemia salina* larvae were obtained from Department of Veterinary Pharmacology, Faculty of Veterinary Medicine, University of Nigeria, Nsukka.

## 5. Methods

### 5.1. Plant Collection and Preparation 

The fresh leaves and stem bark of *Lannea barteri* were collected from Benue State, Nigeria in the months of March and April by Mr. Alfred Ozioko of International Centre for Drug Discovery and Development and the specimen voucher (Voucher No: 096) deposited at the Centre’s herbarium. The plant materials were dried under shade, and subsequently pulverized into powder for future use.

### 5.2. Distillation of Solvents Used for Extraction and Fractionation

All extraction solvents were distilled to their pure states according to their specific boiling points: 65°C (methanol), 39.8°C (dichloromethane (DCM)), 59°C (ethyl acetate) and 69°C (n-hexane).

### 5.3. Cold Extraction of the Plant Materials

About 2 g each of the powdered material were subjected to successive cold extraction by using the following solvents in increasing order of polarity; DCM, methanol and water. The powdered material was soaked in about 100 mL solvent in a conical flask and then sonicated for 10 min in an ice pack to break the cell wall of the plant biomass. Afterwards, the flask was placed on a magnetic stirrer for 180 min at 250 rpm. The content of the flask was centrifuged at 3500 rpm for 10 min and the supernatant filtered into a round bottom flask. The extraction process was repeated two more times after which the filtrates were pooled together and evaporated by using a vacuum rotary evaporator in a fume hood according to the boiling pressure of the solvent: 880 mbar (DCM), 337 mbar (methanol) and 72 mbar (water) at 50°C. After extraction with each solvent, the marc was dried overnight in a fume hood before the next solvent was used until the last solvent. The concentrated extracts yielded cold (DCM) leaf (DLEc) and stem bark (DSbEc) extracts, cold methanol leaf (MLEc) and stem bark (MSbEc) extracts and cold water leaf (WLEc) and stem bark (WSbEc) extracts which were weighed, yield in (%) calculated and stored in the refrigerator for future use. 

The extracts were subjected to anticancer screening by the crystal violet cell proliferation assay as a bioactivity guide (see below).

### 5.4. Sohxlet (hot) Extraction of the Plant Materials

About 10 g of the plant material was extracted successively with these solvents in the increasing order of polarity according to their boiling temperatures 39.8°C (DCM), 65°C (methanol), and 100°C (water) in continuous extraction for 12 h by using a Sohxlet extraction apparatus. After extraction with each solvent, the marc was dried overnight in a fume hood before the next solvent was used until the last solvent. The extracts were concentrated using rotary evaporator as described in Section 5.3 to yield hot DCM leaf (DLEh) and stem bark (DSbEh) extracts, hot methanol leaf (MLEh) and stem bark (MSbEh) extracts and hot water leaf (WLEh) and stem bark (WSbEh) extracts. These extracts were weighed, yield in (%) calculated and stored in the refrigerator for future use. 

The extracts were subjected to anticancer screening by the crystal violet cell proliferation assay as a bioactivity guide (see below).

### 5.5. Thin Layer Chromatography (TLC) of the Most Active Extract

The DSbEc and DLEc which exhibited the greatest antiproliferative activity were subjected to TLC by using 1:1 ratio of analytical grade n-hexane and ethyl acetate and the retention factors were calculated [24].

### 5.6. Column Chromatography of DLEc and DSbEc with Gradient Elution Method 

The DLEc and DSbEc were subjected to column fractionation on 60 H 70–230 mesh silica gel by using gradient elution method with these respective solvent combination ratios (9:1 (200 mL), 1:1 (200 mL), 2:8 (200 mL)) of analytical grade n-hexane and ethyl acetate; and lastly 100% methanol (100 mL). The resulted fractions (1A-K from DLEc and 2A-2J from DSbEc) were further subjected to primary screening with the crystal violet cell proliferation assay. The seven fractions (1E-K) from DLEc and six fractions from DSbEc (2D-I) that gave the lowest T/C _corr_. (%) values were selected for secondary screening using crystal violet cell proliferation assay and determination of IC_50_. The fractions were then subjected to HPLC analysis.

### 5.7. High Performance Liquid Chromatographic (HPLC) Separation

The cytotoxic fractions (50 mg/mL) mentioned in Section 5.6 were subjected to reversed phase HPLC analysis with the following conditions: 10 µL were injected onto a 250 × 4 mm Nucleodur PolarTec (5 µm) column (Macherey-Nagel, Dueren, Germany) held at 30°C and eluted with a solvent mixture containing 10 mM (pH =3.3) phosphate buffer (PB) and acetonitrile (ACN) at a flow rate of 0.7 mL/min according to the following program:: – 80:20PB/ACN for 5 min, linear gradient to 20:80 PB/ACN over 20 min, 20:80 PB/ACN for 10 min. Detection was done with a diode array UV/vis detector set to scan wavelengths between 210–600 nm and monitoring at 250 nm for a period of 35 min.

### 5.8. Phytochemical Analysis of DLEc and DSbEc

The DLEc and DSbEc were screened for the presence of bioactive components following the methods of [25,26].

### 5.9. BiologicalStudies

#### 5.9.1. Crystal Violet cell Proliferation Assay

The crystal violet cell proliferation assay was used as a bioactivity guide in both primary and secondary screening assays as previously described [27,28]. To prepare the 96–well microtiter plates, the cell cultures were first treated with trypsin-EDTA to obtain a cell suspension. Then, 100 µL of the cell suspensions were seeded at a density of 1000 cells per well for each cancer cell line. The plates were incubated for 24 h before treatment with the extract samples. Following treatment (see below), the cells were incubated 96 h, cell growth was stopped by replacing the culture medium with 1% glutaraldehyde buffered saline (GBS) for 20 min and then stored under Dulbeccos’s buffer solution. Later, the buffer solution was removed and the cells were stained with 0.02% crystal violet in deionized water (100 µl per well) for 30 min. Thereafter, the excess dye was discarded and the plates washed in water for 30 min. The cell–bound dye was re–dissolved with 70% ethanol (100 µl per well) and the plates were placed on the microtiter shaker for 2 h before the optical density (OD) was read at λ = 570 nm with a microtiter plate reader. The corrected percent growth values, e.g., Treated versus Control corrected percentage (T/C) _corr._ (%) was calculated with the equation:

(T/C) _corr._ (%) = (OD_T_ – OD_C,0_)/(OD_C_ – OD_C,0_) × 100

where OD_T_ is the mean OD of the treated cells, OD_C_ the mean OD of the controls, and OD_C,0_ the mean OD of seeded cells at the time the drug was added. 

The IC_50_ (half maximal inhibitory concentration) values were estimated by linear least – square regression of the (T/C) _corr._ (%) by using MS Excel^®^ function trend values versus the logarithm of the concentration of extract. The concentration that showed (T/C) _corr._ values between 10% and 90% were used in the calculation. The reported IC_50_ values are the average of 3 independent experiments. The IC_50_ value indicated the potency of the extract and equaled the amount of complex that inhibited the cell growth by 50%. The lower the IC_50_ value the greater the potency of the extract.

##### Primary Screening

Cells were treated with stock solution of the extracts dissolved in DMSO at 50 mg/mL; a 10 µL of the stock solution was added to 5.0 mL of RPMI medium containing 10% FCS and 1% penicillin/streptomycin solution, of which 100 µL of the solution was added to each well to give final concentrations of 50 µg/mL extract mass.. One plate was left untreated for each cell line and this served later as the control (C) at time zero (0) (C, 0) when results were obtained. The plates at time zero were stopped as previously described above. Treated cells were returned to the incubator for 96 h at 37°C and stored after fixation with GBS in the refrigerator at 4°C until required [27]. The T/C _corr_. values for the extracts were calculated as described above.

##### Secondary Screening

Two-fold serial dilutions of the 50 mg/mL stock solution were made by diluting with DMSO to obtain five stock dilutions. The stock solution was diluted 1:500 with the culture medium and 100 µl were added to cells in 96-well plate as triplicates starting with the least concentration to the highest concentration [27]. The plates were returned to the incubator for 96 h at 37°C and the plates at time zero were stopped with glutaraldehyde fixation. After incubation period, treated cells were handled as described above and IC_50_ values of the extracts estimated.

#### 5.9.2. Brine Shrimp Toxicity Assay (BSCA)

The toxic effect of DLEc and DSbEc on brine shrimp larvae was measured by using a previously reported method [29,30].

#### 5.9.3. Determination of Mechanism of Cytotoxic Action of Most Active Fractions on SiSo Cancer Cell Line 

Fractions 1H, 1I and 2E, 2F were selected for apoptotic and cell cycle analysis with the SiSo cell line.

##### Apoptosis Measurement

The induction of apoptosis of the fractions on SiSo cell line was determined by using an Annexin-V/propidium iodide double staining, flow cytometric method as described previously [31].

##### Cell Cycle Analysis: Propidium Iodide (PI) Staining

The ability of the fractions to arrest the cell cycle progression in SiSo cell line was done by staining cells with propidium iodide and measuring the phases of the cell cycle by flow cytometry as described previously [32].

**Scheme 1:**
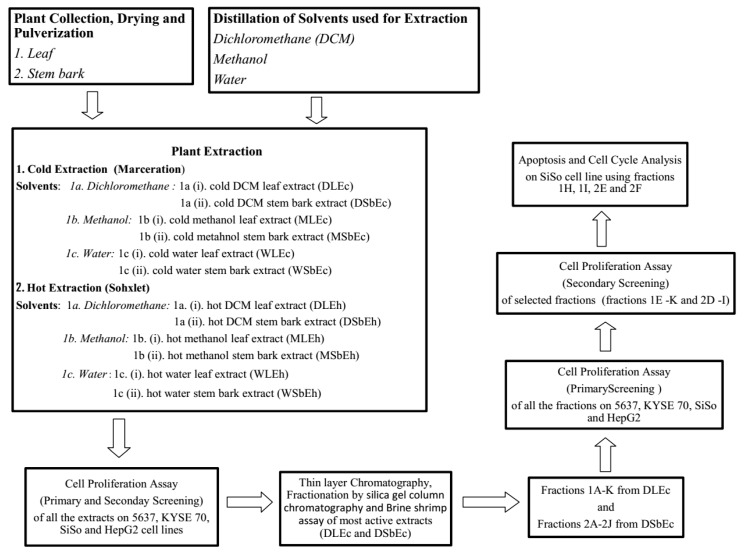
Flowchart summary of the extraction, fractionation and testing procedures.

A summary of the extraction, fractionation and testing procedures is shown in Scheme 1.

## Figures and Tables

**Table 1:** Yield of *L. barteri* leaves and stem bark extracts.

Extract	Yield	
	mg	%
DLEc	40.1	1.86
DSbEc	46.1	1.15
DLEh	235.2	2.35
DSbEh	216.1	2.37
MLEc	195.0	9.75
MSbEc	78.5	3.93
MLEh	748.7	17.49
MSbEh	912.0	10.03
WLEc	90.4	4.52
WSbEc	47.0	2.35
WLEh	718.8	7.17
WSbEh	227.3	2.27

DLEc—cold dichloromethane (DCM) leaf extract, DSbEc—cold DCM stem bark extract, DLEh—hot DCM leaf extract, DSbEh—hot DCM stem bark extract, MLEc—cold methanol leaf extract, MSbEc—cold methanol stem bark extract, MLEh—hot methanol leaf extract, MSbEh—hot methanol stem bark extract, WLEc—cold water leaf extract, WSbEc—cold water stem bark extract, WLEh—hot water leaf extract and WSbEh—hot water stem bark extract.

**Table 2:** **TLC** retention factor (R_f_) values of the most active extracts.

Solvent Ratio (n-hexane: ethyl acetate)	DLEc	DSbEc
	R_f_	
9:1	0.25	0.30
8:2	0.38	0.32
7:3	0.47	0.50
6:4	0.50	0.55
5:5	0.68	0.70
4:6	0.44	0.38
3:7	0.40	0.30
2:8	0.26	0.28
1:9	0.22	0.25

DLEc = cold DCM leaf extract; DSbEc = cold DCM stem bark extract.

**Table 3:** Yield of fractions from chromatography of cold DCM leaf and stem bark extracts.

Fractions	Pooled Samples	Weight (mg)	Yield (%)
1A	8–13	3.30	0.66
1B	14–20	15.50	3.10
1C	21–28	27.80	5.56
1D	29–37	13.40	2.68
1E	38–42	113.70	22.74
1F	43–47	31.70	6.34
1G	48–56	13.60	2.72
1H	57–85	14.90	2.98
1I	86–102	7.80	1.56
1J	103–187	41.00	8.20
1K	188–192	39.40	7.00
			
2A	1–7	8.20	1.64
2 B	8–24	9.30	1.86
2 C	25–40	13.00	2.60
2 D	41–44	29.60	5.92
2 E	45–51	48.20	9.64
2 F	52–61	69.80	13.96
2 G	62–77	59.50	11.90
2 H	78–105	54.10	10.82
2 I	106–109	84.00	16.80
2 J	110–140	8.80	1.76

(1A–K) and (2A–J) represent fractions from DLEc and DSbEc respectively.

**Table 4:** Antiproliferative activity T/C _corr_. (%) of extracts (50 µg/mL) on 5637, KYSE 70 and SiSo cell lines in primary screening.

Extract		Cell Line	
	5637	KYSE 70	SiSo
	T/C _corr_. (%)		
DLEc	14.76	5.83	4.62
DSbEc	7.82	3.03	2.56
DLEh	2.01	5.18	4.21
DSbEh	-3.12	4.63	2.67
MLEc	1.77	>50	20.95
MSbEc	3.64	>50	24.33
MLEh	>50	>50	>50
MSbEh	>50	>50	>50
WLEc	>50	>50	>50
WSbEc	>50	>50	>50
WLEh	>50	>50	>50
WSbEh	>50	>50	>50

Stock solution concentration = 50 µg/mL DMSO, *n* = 2; DLEc—cold DCM leaf extract, DSbEc—cold DCM stem bark extract, DLEh—hot DCM leaf extract, DSbEh—hot DCM stem bark extract, MLEc—cold methanol leaf extract, MSbEc—cold methanol stem bark extract, MLEh—hot methanol leaf extract, MSbEh—hot methanol stem bark extract, WLEc—cold water leaf extract, WSbEc—cold water stem bark extract, WLEh—hot water leaf extract and WSbEh—hot water stem bark extract; T/C _corr_.—treated versus control corrected.

**Table 5:** IC_50_ of extracts on KYSE 70 and SiSo cell lines in secondary screening.

Extract	Cell Line	
	KYSE 70	SiSo
	IC_50_ (µg/mL)	
DLEc	11.03 ± 2.68	12.06 ± 8.39
DSbEc	2.26 ± 0.92	6.37 ± 2.15
DLEh	12.23 ± 3.12	10.68 ± 2.36
DSbEh	5.28 ± 3.43	5.62 ± 3.00
MLEc	>50	11.01 ± 4.04
MSbEc	>50	23.85 ± 9.41
MLEh	>50	20.93 ± 4.09
MSbEh	>50	>50

Stock solution concentration = 50 µg/mL DMSO for DLEh, DSbEh, MLEc, MSbEc, MLEc and MSbEh, 25 µg/mL DMSO for DLEc and DSbEc. *n* = 3; DLEc—cold DCM leaf extract, DSbEc—cold DCM stem bark extract, DLEh—hot DCM leaf extract, DSbEh—hot DCM stem bark extract, MLEc—cold methanol leaf extract, MSbEc—cold methanol stem bark extract, MLEh—hot methanol leaf extract, MSbEh—hot methanol stem bark extract, WLEc—cold water leaf extract, WSbEc—cold water stem bark extract, WLEh—hot water leaf extract and WSbEh—hot water stem bark extract.

**Table 6:** Antiproliferative activity as T/C _corr._ (%) of fractions (50 µg/mL) on 5637, KYSE 70, SiSo and HepG2 cell lines.

Fraction		Cell line		
	5637	KYSE 70	SiSo	HepG2
	T/C _corr_. (%)			
1A	33.67	85.26	108.84	99.21
1B	3.06	62.12	100.00	20.58
1C	3.06	69.29	116.02	44.33
1D	−9.18	61.57	−10.50	37.99
1E	-11.22	−0.22	−4.18	−7.39
1F	−1.02	−2.22	−4.50	−10.95
1G	−9.66	−2.95	−6.75	−12.40
1H	−6.90	−4.15	−6.59	14.25
1I	−7.38	−4.04	−6.27	14.12
1J	−8.18	−3.82	−6.11	14.38
1K	15.54	−4.42	−2.73	10.55
2A	22.94	71.51	23.79	83.51
2B	−14.94	51.64	21.57	48.15
2C	−16.91	12.01	6.11	−1.58
2D	−16.21	−4.91	−0.07	−14.64
2E	−15.01	−5.13	−7.24	−14.51
2F	−17.01	−4.80	−7.07	−14.51
2G	−13.91	−5.02	−6.43	−13.98
2H	−8.91	−4.80	−6.01	−14.64
2I	−13.17	−4.04	−5.95	−11.21
2J	−9.34	62.55	22.51	10.82

**Table 7:** Toxic effect of extracts on brine shrimp.

Treatment	Dose (µg/mL)	Alive	Dead	Dead (%)
**DLEc**	10	29	1	3.3
	100	25	5	16.7
	1000	26	4	13.3
**10% Tween 80**	-	30	-	0
**DSbEc**	10	17	13	43.0
	100	11	19	63.3
	1000	10	20	66.7

The estimated LC_50_ were 5649.20 µg/mL (DLEc) and 124.78 µg/mL (DSbEc); EC_50_ were 564.92 µg/mL (DLEc) and 12.48 µg/mL (DSbEc) and therapeutic index (TI) was 9.99 for both. LC_50_ and EC_50—_lethal and effective concentrations at 50% population respectively, DLEc—cold DCM leaf extract, DSbEc—cold DCM stem bark extract, *n* = 30.

## Supplementary Materials

The RP-HPLC Chromatograms of active fractions from *Lannea barteri* Oliv. (Anacardiaceae) cold DCM leaf (fractions1-K) and stem bark (fractions 2D-2J) extracts. (Figures S1–S13: Chromatogram of fractions 1E-1K and 2D-2I. Chrom Type: Integrated Chromatogram between wavelengths 240 to 260 nm respectively). DOI: 10.5277/molecule: 171819 link: https://molecule.org/record/202122.
