# Supplementary material for: Pharmacological Evaluation of the Anticancer Activity of Extracts and Fractions of Lannea barteri Oliv. (Anacardiaceae) on Adherent Human Cancer Cell Lines
Source: Molecules. 2020 Feb 14;25(4):849. doi: 10.3390/molecules25040849 (PMC7070605; doi:10.3390/molecules25040849)
Supplement: Supplementary file 1 [file molecules-25-00849-s001.pdf]

Supplementary Material

DOI: 10.5277/molecule:171819 link:https://molecule.org/record/202122

Pharmacological Evaluation of the Anticancer Activity of Extracts and Fractions of *Lannea barteri* Oliv. (Anacardiaceae) on Adherent Human Cancer Cell Lines

Florence N. Mbaoji[1,2,*], Steven Behnisch-Cornwell [2], Adaobi C. Ezike[1], Chukwuemeka S. Nworu[1] and Patrick J. Bednarski[2,*]

[1] Department of Pharmacology and Toxicology, Faculty of Pharmaceutical Sciences, University of Nigeria, Nsukka, PMB 410001, Enugu State, Nigeria

[2] Institute of Pharmacy, Pharmaceutical and Medicinal Chemistry, University of Greifswald, 17487 Greifswald, Germany;

The RP-HPLC chromatograms of active fractions from *Lannea barteri* Oliv (Anacardiaceae) cold DCM leaf (fractions 1E-1K) and stem bark (fractions 2D-2J) extracts

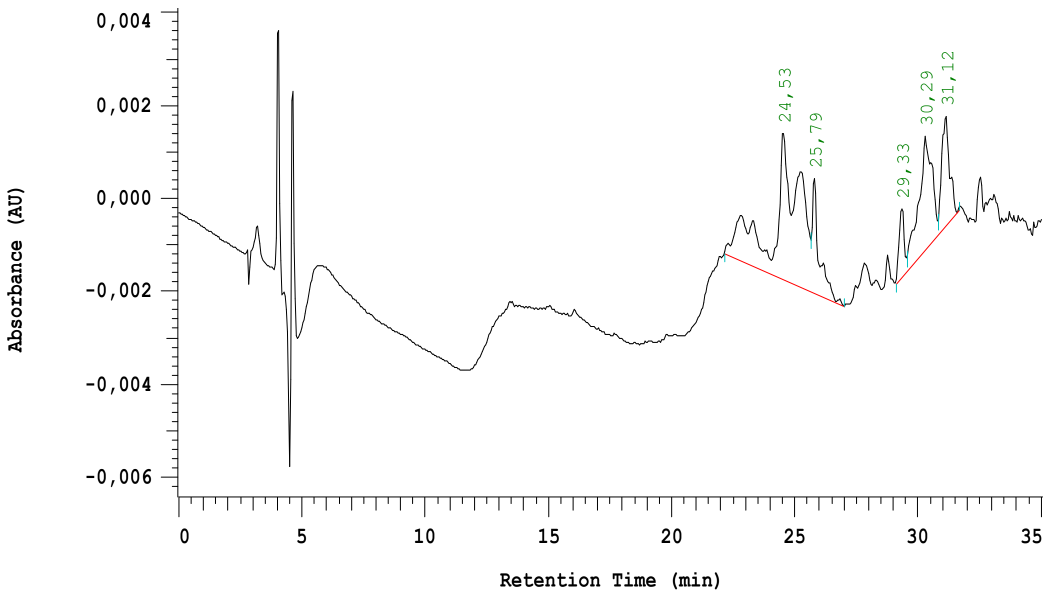

Figure S1: Chromatogram of fraction 1E. Chrom Type: Integrated Chromatogram between wavelengths 240 to 260 nm

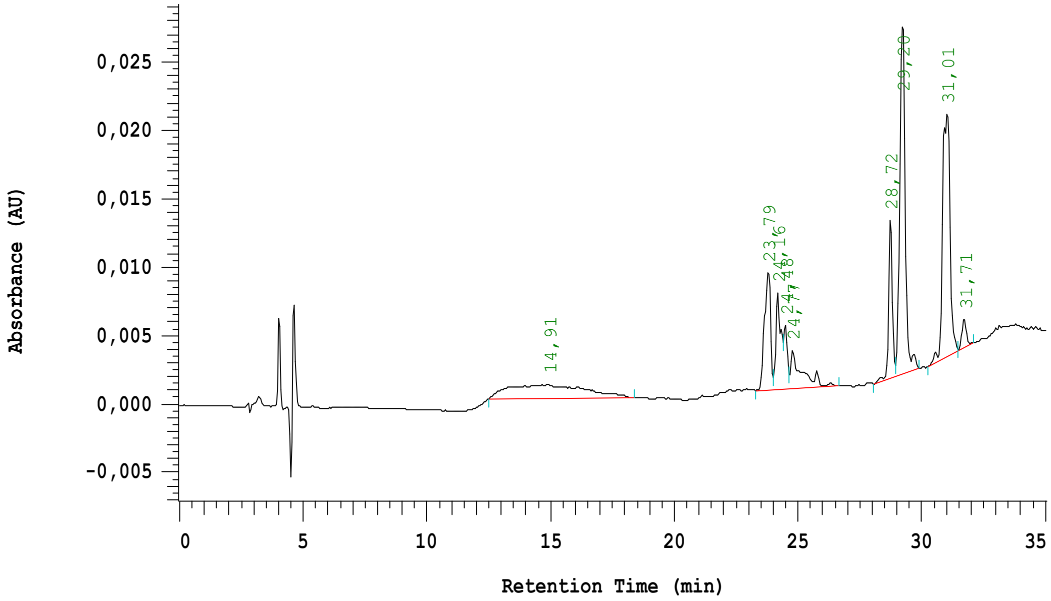

Figure S2: Chromatogram of fraction 1F. Chrom Type: Integrated Chromatogram between wavelengths 240 to 260 nm

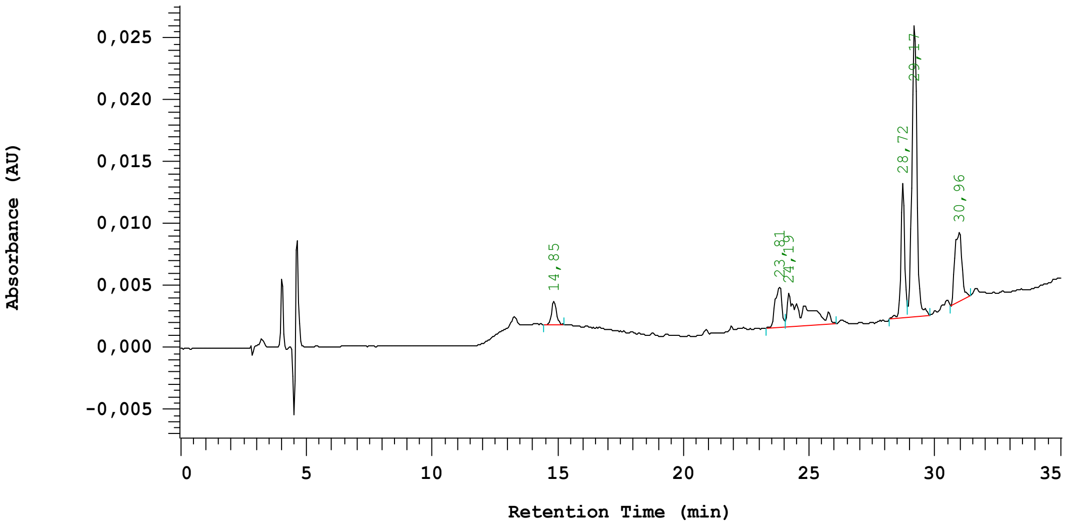

Figure S3: Chromatogram of fraction 1G. Chrom Type: Integrated Chromatogram between wavelengths 240 to 260 nm

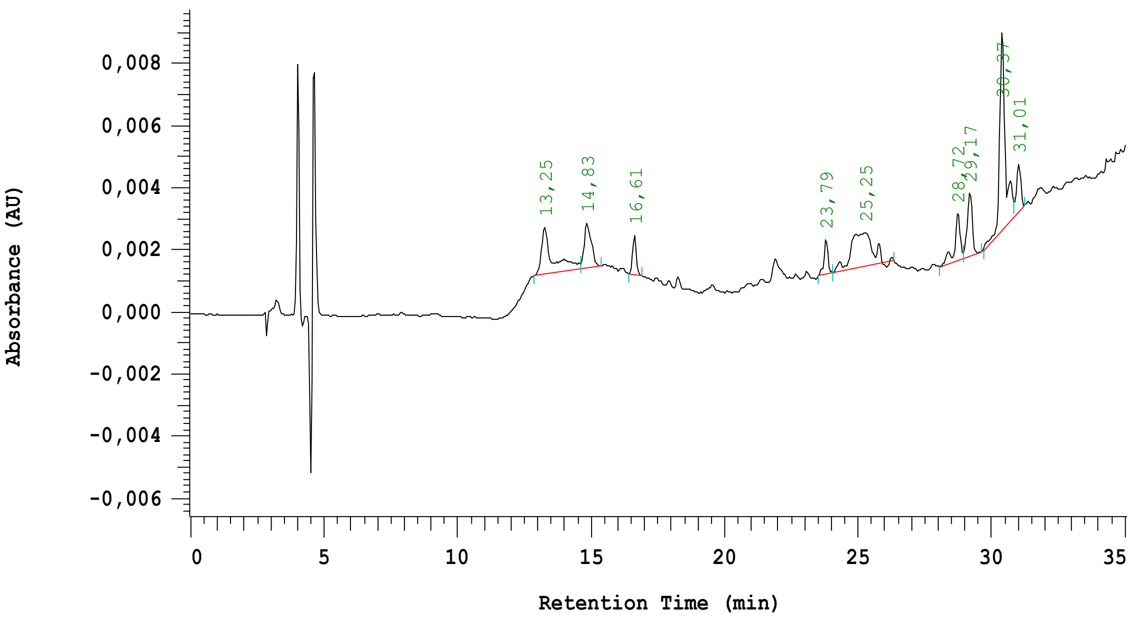

Figure S4: Chromatogram of fraction 1H. Chrom Type: Integrated Chromatogram between wavelengths 240 to 260 nm

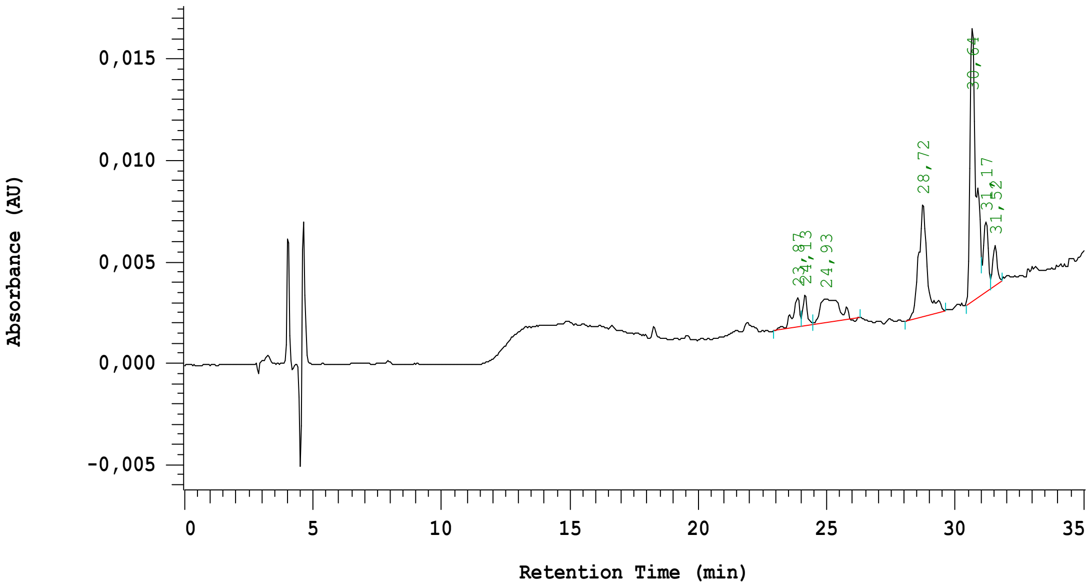

Figure S5: Chromatogram of fraction 1I. Chrom Type: Integrated Chromatogram between wavelengths 240 to 260 nm

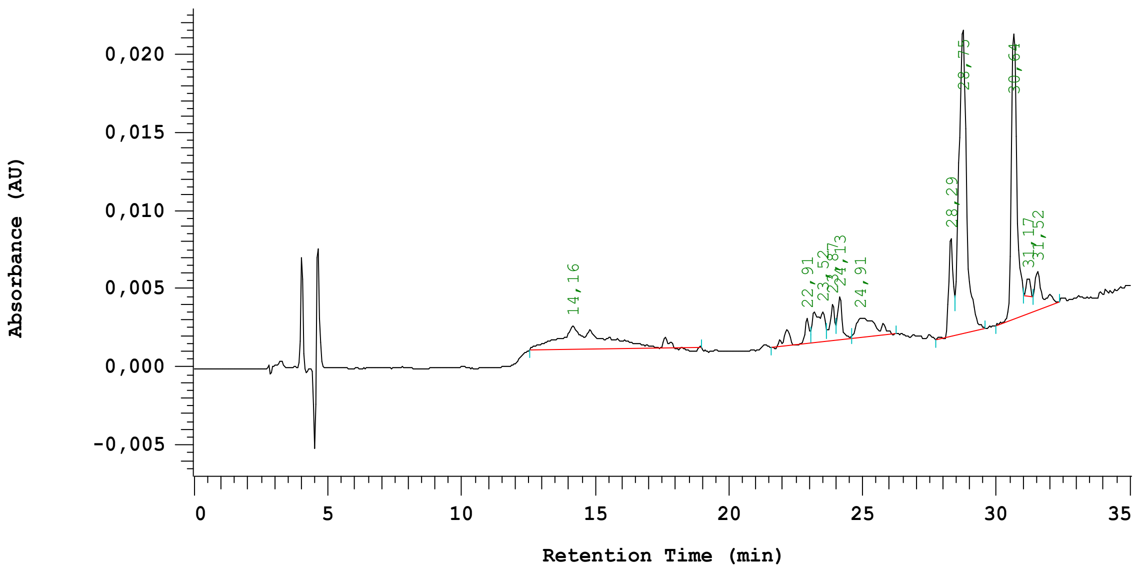

Figure S6: Chromatogram of fraction 1J. Chrom Type: Integrated Chromatogram between wavelengths 240 to 260 nm

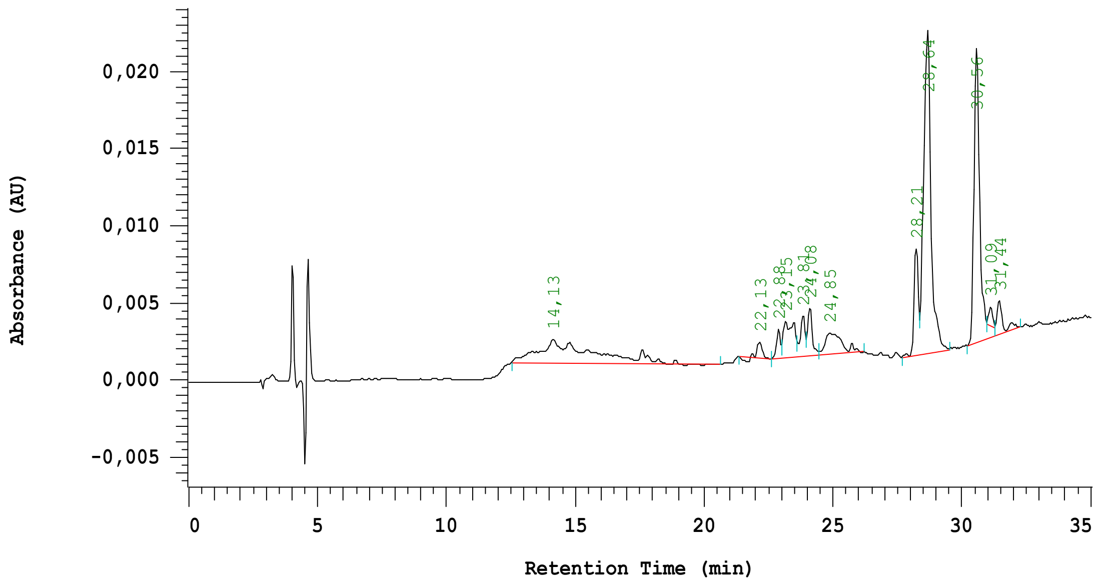

Figure S7: Chromatogram of fraction 1K. Chrom Type: Integrated Chromatogram between wavelengths 240 to 260 nm

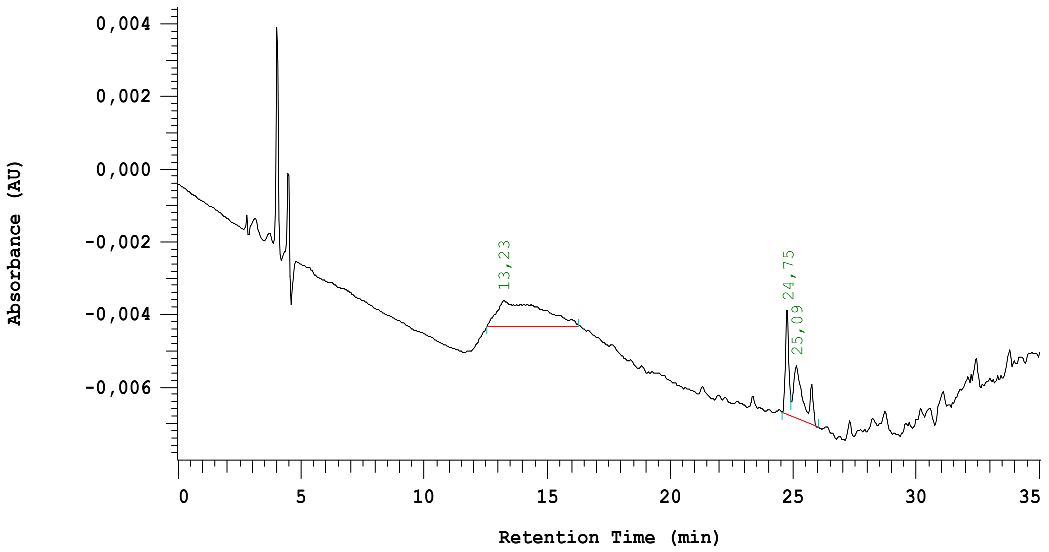

Figure S8: Chromatogram of fraction 2D. Chrom Type: Integrated Chromatogram between wavelengths 240 to 260 nm

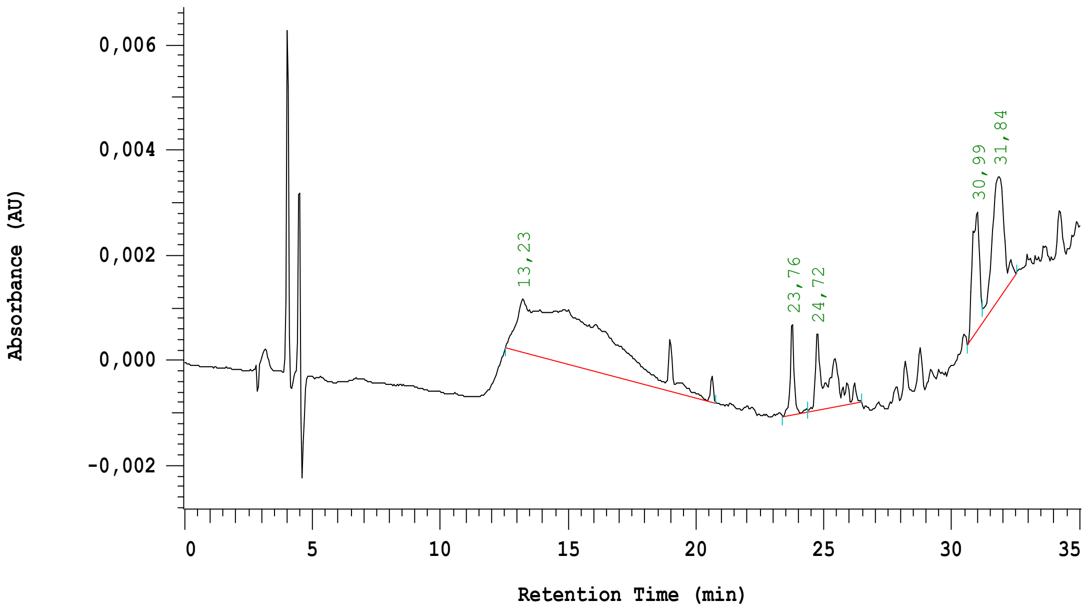

Figure S9: Chromatogram of fraction 2E. Chrom Type: Integrated Chromatogram between wavelengths 240 to 260 nm

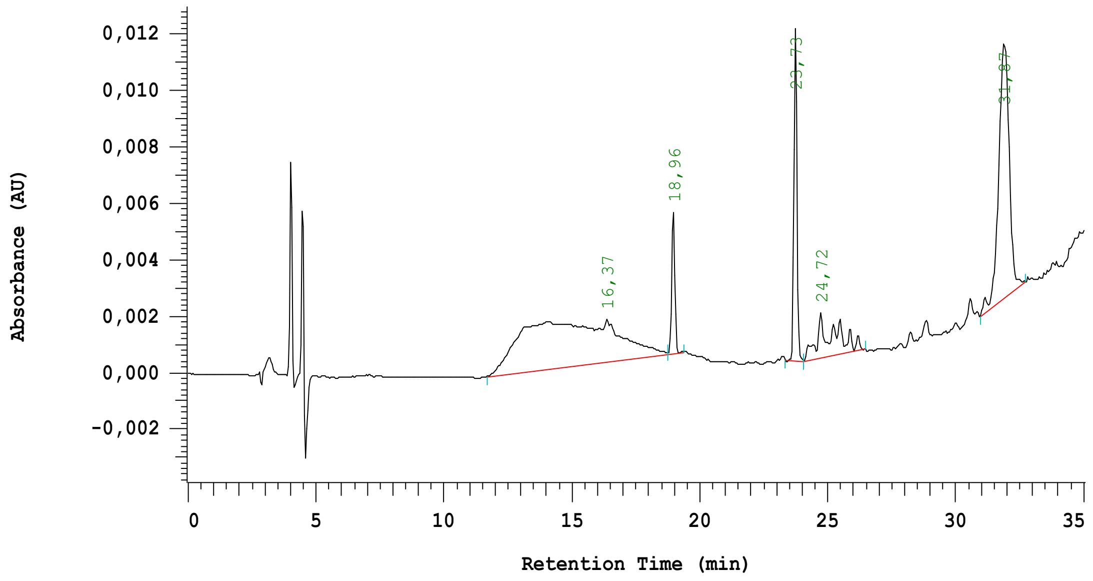

Figure S10: Chromatogram of fraction 2F. Chrom Type: Integrated Chromatogram between wavelengths 240 to 260 nm

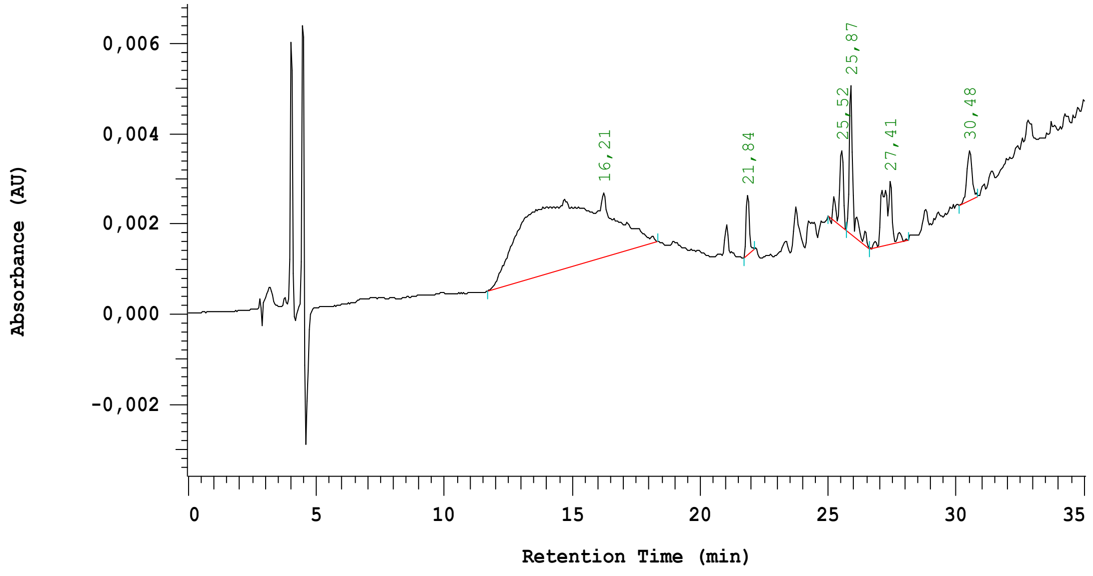

Figure S11: Chromatogram of fraction 2G. Chrom Type: Integrated Chromatogram between wavelengths 240 to 260 nm

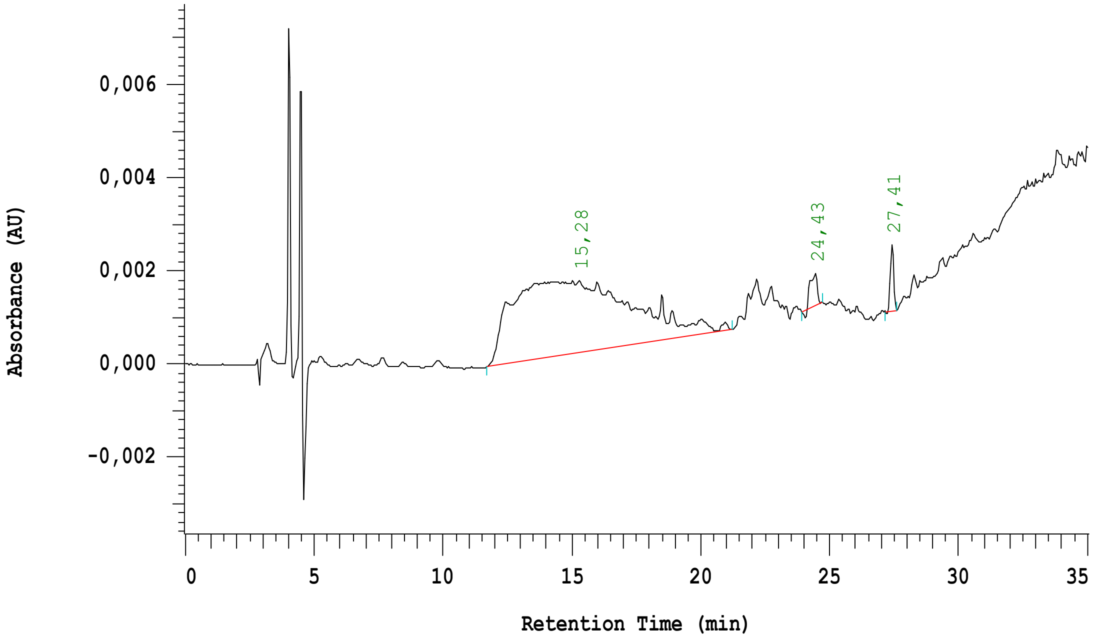

Figure S12: Chromatogram of fraction 2H. Chrom Type: Integrated Chromatogram between wavelengths 240 to 260 nm

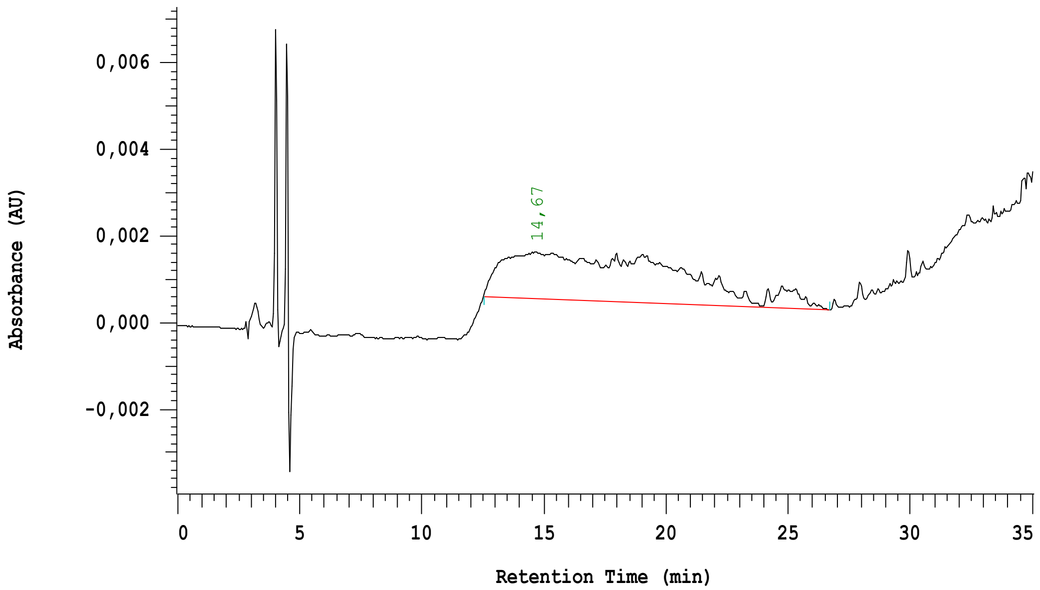

Figure S13: Chromatogram of fraction 2I. Chrom Type: Integrated Chromatogram between wavelengths 240 to 260 nm
